# The Arrest Is a Trajectory, Not a Snapshot: Rethinking Point-of-Care Ultrasound During Cardiac Arrest

**DOI:** 10.7759/cureus.114500

**Published:** 2026-08-13

**Authors:** Chetla Rakesh, Naveen Mohan

**Affiliations:** 1 Emergency Medicine, Amrita Institute of Medical Sciences and Research Centre, Kochi, IND

**Keywords:** cardiac arrest, cardiac standstill, clinical decision-making, emergency medicine, point-of-care ultrasound, prognosis, pulseless electrical activity, resuscitation, return of spontaneous circulation, serial ultrasound

## Abstract

Point-of-care ultrasound (POCUS) is now routine during resuscitation, and clinicians may interpret a single intra-arrest finding, such as cardiac standstill, pseudo-pulseless electrical activity (PEA), or organized motion, as a fixed description of the patient’s physiological state. This perspective argues that this assumption deserves scrutiny. The physiological state during cardiac arrest may evolve over time; cardiac mechanical activity may follow a trajectory that unfolds over minutes or a longer resuscitation. Published intra-arrest POCUS protocols generally define when and how imaging should be performed during brief interruptions in cardiopulmonary resuscitation (CPR) but do not provide a standardized framework for interpreting findings according to elapsed time from arrest recognition. Accordingly, the same ultrasound finding may represent different points in the resuscitation. Existing prognostic and diagnostic reviews of intra-arrest ultrasound have documented substantial variation in the timing of image acquisition without resolving what that variation means for interpretation. We propose that ultrasound findings are better interpreted as points along a trajectory than as isolated, static observations. This may provide additional temporal context for interpreting prognostic findings and help avoid overinterpretation of a single early intra-arrest observation. We translate this concept into five practical considerations: recording elapsed arrest time at acquisition, avoiding premature futility judgments from early standstill, interpreting pseudo-PEA as a potentially transitional state, considering repeat scanning where feasible without prolonging interruptions in high-quality CPR, and using ultrasound as an adjunct to established clinical assessment and resuscitation decision-making. This framework is conceptual rather than a demonstrated physiological model; available evidence does not currently establish whether observed differences reflect distinct physiological phenotypes, timing effects, or both. This hypothesis could be evaluated through prospective studies that record the timing of clinically obtained ultrasound examinations and, where serial imaging is clinically indicated, assess changes over the course of resuscitation without prolonging interruptions in CPR. Studies incorporating standardized acquisition timing and blinded interpretation of stored recordings could help determine whether acquisition timing contributes to the interpretation and prognostic value of intra-arrest ultrasound findings. Until then, clinicians should interpret intra-arrest ultrasound with attention to when a finding occurred, not only what it showed.

## Editorial

A point-of-care ultrasound (POCUS) image obtained during cardiac arrest can provide direct visual information about cardiac mechanical activity that a pulse check cannot. However, ultrasound does not replace pulse assessment for determining return of spontaneous circulation (ROSC), and current resuscitation guidance continues to emphasize rapid pulse assessment while minimizing interruptions in chest compressions. The visual nature of ultrasound may nevertheless create a strong impression of objectivity, making a single image of cardiac standstill particularly susceptible to being interpreted as more definitive than the surrounding clinical context warrants. A single clip showing cardiac standstill is one observation obtained during an evolving resuscitation, rather than a complete description of the patient's physiological state.

Cardiac arrest is clinically defined by the absence of effective cardiac output, but the physiological state during resuscitation can evolve over time. Cardiac mechanical activity may vary in its presence and intensity during an arrest, potentially changing as resuscitation progresses. We hypothesize that these changes may represent a temporal trajectory rather than a series of entirely distinct physiological states. Thus, two patients classified as having cardiac standstill by the same ultrasound assessment may have been imaged at different points in their resuscitation, for example, early versus later after arrest recognition. The temporal difference between early and prolonged arrest is clinically recognized; the unresolved question is whether elapsed arrest time also modifies the prognostic meaning assigned to a specific ultrasound finding. Published intra-arrest POCUS approaches specify when imaging should be performed in relation to CPR and rhythm-check pauses, but the prognostic literature has used variable timing of echocardiographic assessment, and whether this timing influences the prognostic meaning of a given ultrasound finding remains uncertain [1,2].

This raises a practical question for clinicians: how should a single ultrasound finding be read when the physiology it describes is still in motion? The premise of this perspective is that ultrasound findings are best interpreted as points along a trajectory rather than as isolated, static observations and that this reframing changes how five common clinical decisions should be approached.

Why a snapshot can mislead

Cardiac standstill, pseudo-pulseless electrical activity (pseudo-PEA), and organized cardiac motion are findings observed at particular points during resuscitation. Existing intra-arrest POCUS approaches provide temporal structure around CPR and rhythm-check pauses. For example, the International Sonography in Hypotension and Cardiac Arrest (SHoC) consensus specifies that core cardiac views should be obtained during the rhythm-check pause without prolonged interruption of chest compressions, and its checklist incorporates review of images during CPR followed by clinical reassessment [1]. This diagnostic role is distinct from the prognostic use of cardiac ultrasound to assess the likelihood of ROSC or survival, for which the evidence remains less established.

Across the prognostic literature, however, the timing of echocardiographic assessment has varied substantially, including initial, repeated, subsequent, or unspecified examinations [2]. Thus, the issue is not whether ultrasound can be repeated during resuscitation, but whether the temporal context of an individual finding is consistently incorporated into its prognostic interpretation. A recent single-center cohort of in-hospital cardiac arrests (IHCAs) found substantially higher odds of ROSC among patients with pseudo-PEA than those with true PEA, but whether this difference is modified by the timing of ultrasound acquisition remains uncertain [3].

Five practical principles for clinicians

Record Elapsed Arrest Time Alongside Each Ultrasound Finding

A technical timestamp identifies when an image was acquired but does not necessarily indicate where that observation occurred in the course of resuscitation. Where the time of arrest recognition can be established, recording elapsed arrest time alongside the ultrasound finding would provide the temporal context needed to examine whether the prognostic meaning of a finding varies during resuscitation.

Consider the Timing of Standstill Within the Overall Clinical Assessment

Absence of cardiac motion is associated with a lower likelihood of survival, but it does not have sufficient accuracy to serve as a stand-alone determinant of outcome or termination of resuscitation [4]. Accordingly, standstill should remain one component of the overall clinical assessment; our proposed addition is that the timing of its acquisition may also deserve consideration when judging its prognostic significance.

Interpret Pseudo-PEA as a State That May Still Be in Transition

For this perspective, pseudo-PEA refers to organized electrical activity in the absence of a palpable pulse, with coordinated cardiac mechanical activity demonstrated on POCUS. In a recent single-center cohort of IHCA, pseudo-PEA was specifically defined as coordinated cardiac motion on POCUS, whereas absent cardiac activity or disorganized cardiac motion, including fibrillatory movement, was classified as true PEA for analysis [3]. The observed association between pseudo-PEA and improved ROSC and longer-term survival should therefore be interpreted as a prognostic association rather than as evidence that pseudo-PEA alone determines prognosis or management. Clinically, pseudo-PEA should remain one finding within the broader assessment of the arrest rather than a stand-alone basis for treatment or termination decisions.

Where Feasible, Repeat the Scan Rather Than Relying on One

A repeat ultrasound examination may provide additional information about changes in cardiac mechanical activity during resuscitation that cannot be inferred from a single image. However, whether serial imaging changes clinical management or improves patient outcomes has not been established. When repeat imaging is clinically indicated, it should be incorporated into a subsequent rhythm-check pause without prolonging the interruption in chest compressions or compromising high-quality CPR.

Use Ultrasound as Part of the Overall Resuscitation Assessment

Termination-of-resuscitation rules provide structured criteria for specific cardiac arrest settings but do not encompass the entirety of clinical decision-making. Recent IHCA termination-of-resuscitation models have been developed and validated without incorporating ultrasound findings [5]. This does not imply that ultrasound should be excluded from decisions about continuation or termination of resuscitation.

Rather, when interpreted by clinicians familiar with its limitations and integrated with the patient's overall clinical context, POCUS may contribute to the decision-making process. Its prognostic role remains insufficiently established to support ultrasound findings as a stand-alone criterion for termination, and its use should not prolong rhythm checks or interrupt high-quality CPR.

Testing this framework will require studies that examine ultrasound findings in relation to both elapsed arrest time and the clinical events occurring during resuscitation. A prospective study could record the time of arrest recognition, initiation of CPR, each ultrasound acquisition, and major resuscitation interventions occurring around the examination, including administration of vasoactive drugs and rhythm changes. Rather than acquiring research images at arbitrary fixed intervals, serial images could be analyzed when obtained during clinically indicated rhythm checks, allowing the relationship between elapsed arrest time and changes in cardiac mechanical activity to be examined without introducing additional interruptions in CPR [1]. The prognostic analysis would need to account for the clinical and treatment context surrounding each acquisition, given that the timing of POCUS and resuscitative interventions can confound prognostic interpretation [2]. Blinded review of stored ultrasound recordings could be used to assess the reproducibility of image classification while preserving acquisition timing for subsequent analysis [2]. Retrospective analyses of existing cohorts could provide an initial test of whether the prognostic performance of persistent cardiac standstill differs according to acquisition time [4].

The clinically important question is therefore whether the prognostic performance of persistent cardiac standstill varies with elapsed arrest time and whether a clinically meaningful time point can eventually be identified at which the finding might contribute to termination decisions within the broader clinical assessment. Existing observational studies of serial ultrasound provide a rationale for investigating this question, including studies in which prolonged standstill was associated with high specificity for non-ROSC; however, these findings have not established a validated time threshold for termination of resuscitation [2,4]. Any proposed threshold would require prospective validation and assessment alongside established clinical factors rather than being inferred from ultrasound alone.

A single intra-arrest ultrasound image should therefore be interpreted as one observation within an evolving resuscitation rather than as a definitive prognostic assessment. When serial imaging is clinically appropriate, changes over time may provide additional information that a single examination cannot capture, but whether this improves clinical decision-making or patient outcomes remains unestablished. Future studies should determine whether incorporating acquisition timing and serial changes in cardiac mechanical activity improves prognostic discrimination without compromising high-quality CPR.
